# Protocols for *in situ* continuous monitoring of water relations/potential in soil and leaf

**DOI:** 10.12688/openreseurope.21382.2

**Published:** 2026-05-29

**Authors:** Lei Ding, Marco D'Agostino, Théo Degand, Monica Rothwell, Thomas Dagbert, Valentin Couvreur

**Affiliations:** 1 Earth and life Institute, Université catholique de Louvain, Louvain-la-Neuve, Walloon Region, 1348, Belgium

**Keywords:** Soil water potential; leaf water potential; Teros 21; psychrometer; PSY1; maize; tomato; data analysis

## Abstract

Within the soil-plant-atmosphere continuum, water movement is driven by the water potential gradients between these three domains. To have a comprehensive understanding of such water relations, an examination of how plants respond to variations in soil water availability is required. The methodologies employed for measuring water potential in leaf (Ψ *_leaf_*) and soil (Ψ *_soil_*) have undergone a significant evolution; transitioning from qualitative assessments to the use of high-precision digital sensors over the past few decades. The present protocol aims to provide a comprehensive, step-by-step guide from the germination phase of maize and tomato plants to the installation of two sensors that continuously monitor water potential in the leaf (PSY1 psychrometer) and in the soil (TEROS 21 matric potential sensor). Additionally, we present the code for processing the raw data files in RStudio.

## Plan language summary

In this study, we provide a step-by-step guide for in situ continuous monitoring of water potential in soil and leaves with Teros 21 and PSY1-leaf psychrometers, respectively. Additionally, we present the code necessary for the analysis of the collected data using the RStudio platform.

## Introduction

In the soil-plant-atmosphere continuum (SPAC), water movement is driven by the water potential gradients between the soil (Ψ *_soil_*), the plants (e.g. Ψ *_leaf_*) and the atmosphere (Ψ *_atm_*). The fundamental mechanism underlying this phenomenon in vascular plants, as depicted by the cohesion-tension theory ( Bohm, 1893; Dixon & Joly, 1895), is the transpiration pull. This force in turn generates a negative pressure potential (Ψ *_p_*) in xylem vessels. When water flows across non-selective tissues, such as cell walls and plasmodesmata, it follows the “downhill” gradients of summed Ψ *_p_* and gravitational potential (Ψ *_g_*) (i.e. from high to low potential). When water flows across a semi-permeable membrane, such as cell plasma membranes, a third additive component comes into play: the osmotic potential (Ψ *_o_*). This component, which is a negative pressure, favors water flow towards the side with the highest solute concentration. At the root cylinder scale, radial water flow rate per unit root surface area ( *J* [ *LT* ^−1^]) is generally described by the Fiscus (1975) equation, describing the root as a single “big membrane”. This equation assumes radial water flow rate to be the difference between the soil-root interface and xylem vessels “ *total*” water potential (Ψ *_tot_* [ *ML* ^−1^ *T* ^−2^], expressed in units of pressure), multiplied by the root hydraulic conductivity ( *L _pr_* [ *L* ^2^ *M* ^−1^ *T*]), ( Fiscus, 1975):

$$J=L_{\text{pr}}\times \left(\Psi_{\text{tot},\text{soil}}-\Psi_{\text{tot},\text{leaf}}\right)$$

where the degree of selectivity of the tissues on the radial pathway comes into play as a dimensionless reflection coefficient (σ *_r_*, null when nonselective, one when fully selective) in the calculation of Ψ *_tot_*:

$$\Psi_{\text{tot}}=\Psi_{p}+\Psi_{g}+\sigma_{r}\Psi_{o}$$

Note that for simplicity, the notation Ψ *_p_* is used for both pressure and matric potentials in the soil. Negative Ψ *_p,soil_* expresses the pressure needed to counteract forces retaining water in the unsaturated soil matrix. Additionally, it will be assumed herein that frictions along xylem vessels are negligible for the small plants of this study, and xylem water potential in the root and leaf are equivalent.

Ψ *_leaf_* (herein may refer to water pressure potential or total water potential according to the method of quantification) is a central physiological variable that reflects the water status of plants, and influences critical processes such as photosynthesis, transpiration, and growth ( De Swaef *et al.*, 2022). Ψ *_leaf_* acts as a primary indicator of plant hydration, offering insights into plant responses to environmental stresses, including drought (very negative Ψ *_p,soil_*), salinity, and extreme temperatures *etc.* Understanding the concept of both Ψ *_leaf_* and Ψ *_soil_* is essential for research efforts in plant water relations, drought resistance, and agricultural irrigation management ( Shackel, 2011). Over the past century, the methods for measuring Ψ *_leaf_* and Ψ *_soil_* have evolved significantly, driven by advancements in technology, materials science, and our understanding of soil-plant-water relations ( Ding *et al.*, 2020). This evolution has transformed the field of plant physiology and soil physics, enabling more accurate, efficient, and non-invasive monitoring of plant and soil water status.

In the early stages of research, Ψ *_leaf_* was primarily measured or estimated by visual assessment (such as leaf wilting) and gravimetric techniques (measurement of water loss) ( Veihmeyer & Hendrickson, 1927). In the 1950s and 1960s, direct measurements of Ψ *_leaf_* were achieved by using thermocouple psychrometers (TCP), which typically measure the sum of Ψ *_p_* and Ψ *_o_* ( Boyer & Knipling, 1965; Richards & Ogata, 1958). Concurrently, the Scholander pressure chamber proposed a direct but destructive measurement of (Ψ *_leaf_*) (Ψ *_p_* or Ψ *_tot_*, which are still under debate) ( Scholander *et al.*, 1965). Later, plant physiologists devoted much effort to develop new techniques for the measure of Ψ *_leaf_* with Dew Point Hygrometry ( Banks & Hirons, 2019; Brown & Tanner, 1981). Modern approaches now include infrared thermography ( Cohen *et al.*, 2005), microtensiometers ( Pagay *et al.*, 2014), optical and spectral methods ( Cotrozzi *et al.*, 2017; Wang *et al.*, 2020), and nanosensors ( Jain *et al.*, 2021), allowing for continuous, *in situ*, and highly sensitive monitoring of plant water status. In the context of *in-situ* continuous monitoring of stem or leaf water potential, TCP ( Bellasio *et al.*, 2024; Delval *et al.*, 2024; Haverroth *et al.*, 2025; Jerszurki *et al.*, 2017) and microtensiometers ( Bellasio *et al.*, 2024; Christenson *et al.*, 2024; Delval *et al.*, 2024; Haverroth *et al.*, 2025; Villalobos *et al.*, 2024) have been predominantly utilized in recent years. The microtensiometer is restricted in its application to woody stems; whereas, the TCP can be utilized on both stems and leaves.

Ψ *_soil_* measurement techniques have also evolved from qualitative assessments to high-precision digital sensors ( Bittelli, 2010; Singh *et al.*, 2024; Yao *et al.*, 2025; Yu *et al.*, 2021). Most of the techniques rely on the measurement of the hydraulic equilibrium between soil water and external substances, i.e., solid, liquid and gas ( Campbell & Campbell, 2005; Luo *et al.*, 2022). The techniques are clarified into three main categories, which include solid-based (e.g., filter paper) ( Al-Khafaf & Hanks, 1974; Gardner, 1937), liquid-based (e.g., tensiometer) ( Cassel & Klute, 1986), and vapor-based (e.g., hygrometer) ( Campbell & Campbell, 1974). In field, the most commonly used instruments include piezometers, tensiometers, heat dissipation sensors, dielectric sensors, and TCP. In the laboratory, methods include soil water retention, suction plates, pressure plates apparatus, dew point method, and freezing apparatus ( Bittelli, 2010).

This protocol paper will provide a step-by-step guide for using PSY1 and TEROS 21 sensors for *in situ*, continuous measurements of Ψ *_leaf_* (including pressure potential and osmotic potential) and Ψ *_soil_* (soil matric potential), respectively, on maize and tomato plants. The PSY1 psychrometer (ICT international, Armidale, Australia) is a TCP type sensor that allows the *in situ*, continuous (down to every 10 minutes) measurement of leaf/stem water potential. Teros 21 (Meter group, Washington, United States) is a digital sensor that uses dielectric permittivity changes to estimate matric potential in soil. It works from wet to extremely dry soils, with a detection range of 0 to −100000 kPa and a high precision of 0.1 kPa. As demonstrated in Table 1, a physical interpretation and comparability of TEROS 21 and PSY1 measurements was established. Despite the fact that PSY1 measurements are affected by the soil matric water potential reported by TEROS 21, a direct comparison between the two is not recommended. This is due to the fact that leaf and xylem water potential are also strongly influenced by transpiration-driven declines in pressure potential as well as by osmotic potential.

**Table 1.** Physical interpretation and comparability of TEROS 21 and PSY1 measurements (For more details of Teros 21 and PSY1, please read the user manual from meter group ( https://metergroup.com/products/teros-21/teros-21-support/) and ICT international ( https://ictinternational.com/wp-content/uploads/2023/08/PSY1-Manual-DOC-00087v2.pdf), respectively).

Sensor	Quantity reported	Physical meaning	Remarks
TEROS 21	Soil matric potential	TEROS 21 measures the dielectric permittivity of a solid matrix (porous ceramic discs) to determine its water potential. There is hydraulic equilibrium between the ceramic discs and surrounding soil. So, TEROS 21 requires good hydraulic contact with the surrounding soil for accurate measurements	In salty and dry soils, soil osmotic potential becomes non-negligible, and we need to take the sum of osmotic and matric potential to estimate soil total water potential
PSY1	Leaf/Xylem total water potential	PSY1 measures the vapor pressure in the chamber attached to the water conducting tissue of the plant. It relies on the fact that the liquid phase water in the xylem sets up a vapor pressure equilibrium with the chamber.	It reads both pressure potential and osmotic potential in the measured tissues under thermal and vapor equilibrium between chamber and leaf/xylem, stable temperature, proper installation, no condensation

Additionally, we present user-friendly codes for the analysis and cleaning (e.g. remove outliers and irrelevant values) of the collected data in RStudio.

## Experimental protocols

### Seeds germination and plant growth

Seed resources: *Zea mays L.* (in this example, B104 inbred maize line from previous study ( Ding *et al.*, 2020), referred to as maize later) or *S. lycopersicum var.* Moneymaker (referred to as tomato later, purchased from the market, “Oh! Green”, Belgium, originated from Vilmorin (product number 784)). The accession number for B104 maize is “PI 594047” from the USDA National Plant Germplasm System. The plant passport for tomato seeds is “A *Solanum lycopersicum L.* B FR-RH00867”.

Timing: maize, four weeks; tomato, six weeks.

**Figure 1.**
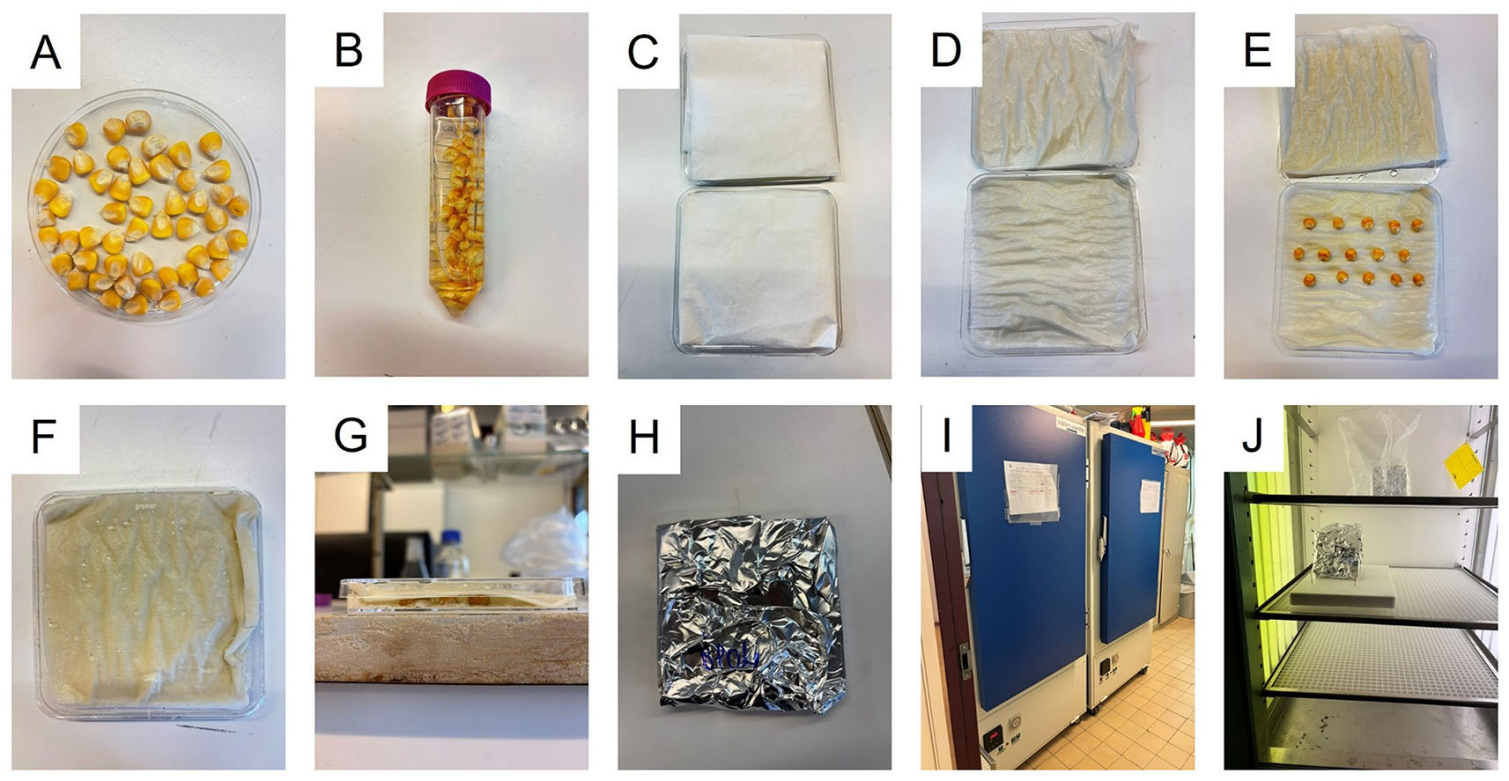
Sterilization and germination of maize seeds. **A**, Prepare maize seeds. **B**, Sterilize seeds. **C**, Prepare the “Sandwich” germination system. **D**, Saturate the paper with demineralized water. **E**, Place seeds on the paper. **F**, Assemble the “Sandwich”. **G**, Side view of the “Sandwich”. **H**, Wrap the “Sandwich” with aluminum foil for dark conditions. **I** and **J**, Germinate the seeds at 25~30°C in a growth chamber.

**Figure 2.**
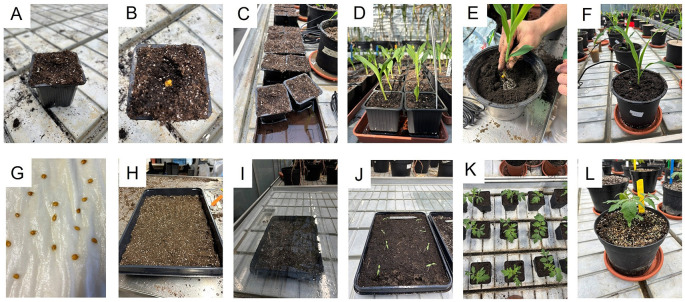
Transplanting and repotting processes. **A**- **F**, maize. **A**, prepare small pots filled with soil. **B**, transfer the germinated seeds into the pot. **C-D **, water the plants and grow them in greenhouse. **E**, transfer the maize seedlings to larger pots (diameter: 15 cm, height: 15 cm) after two weeks. **F**, grow plants in greenhouse. **G-L **, tomato. **G**, tomato seeds after two days germination at 27°C. **H**, prepare a big tray and fill it will soil. **I**, cover the tray with glass to keep moisture of the soil. **J**, remove the glass after 3–4 days. **K**, re-pot the seedling into a larger pot (diameter: 6 cm*6 cm*6 cm [L*W*H]) after 2 weeks. **L**, re-pot the seedling into a larger pot (diameter: 15 cm, height: 15 cm) after 2 weeks.

***1. Sterilization and germination of seeds***

a. Prepare 50 mL of 1/5-strength bleach solution by mixing 10 mL of commercially available bleach (LODA, Bleach 15°, 1815B) with 40 mL demineralized water in a 50 mL falcon tube.
b. Add maize or tomato seeds to the bleach solution for 5 min. Discard the solution and wash five times with demineralized water.
- *Note: To avoid losing the seeds, especially for tomato seeds, a nylon mesh may be used to cover the falcon tube when discarding the bleach solution or water.*
c. In a Petri dish, prepare a “Sandwich” consisting of two ‘layers’ of absorbent paper saturated with water, one on the top and bottom respectively ( Figure 1C–G). Place the seeds with tweezers on the bottom layer and cover them with the other layer of paper, to form the “Sandwich”. Close and wrap the Petri dish with aluminum foil and let it stand vertically at 25~30°C in a climate chamber, growth chamber or oven. Typically, the seeds will germinate after 48 h, at 25~30°C for both maize and tomato. If maize seeds were to be kept in these conditions for more than 48 h, the roots would risk being too long making them difficult to transfer to the soil media in the next step. Instead of using the “Sandwich” technique in “d.”, the seeds may be directly sown in the soil after the sterilization for germination in greenhouse or growth chamber according to the protocol for transplanting ( Figure 2A–C).

***2. Transplanting, repotting and growing the plants in greenhouse***

The plants were grown in the greenhouse of SEFY at UCLouvain ( https://www.uclouvain.be/en/technology-platforms/plant-cultivation-facilities-sefy ). During the experiment periods, the humidity was 50%~70%; the temperature was 21–26°C; and the artificial light was 160~170 μmol.m ^−2^.s ^−1^ at table level. For more information about the greenhouse, please visit the SEFY website.

**Maize**

a. Prepare pots (e.g. 6 × 6 × 6 *cm* [ *L*× *W*× *H*] with drainage holes at the bottom) and fill them with soil. This experiment used seedling & cuttings potting soil ( https://dcm-info.be/fr/pro, DCM, product 1004525), ensuring good water distribution, drainage and allowing the soil to retain its aerated structure ( Figure 2A).
b. If the “Sandwich” technique was used, dig a hole in the soil according to the length of the maize roots and transfer the germinated seeds into the soil (one seed in each pot) ( Figure 2B). Cover the seeds with ~1 cm soil and try to delicately cover the coleoptile (i.e. emerging organ that will give rise to the shoot).
c. Place the pots in a tray and add rainwater to the tray to allow for water uptake by capillary action ( Figure 2C–D). Water the plants regularly to keep the soil moist by the same method.
d. After two weeks, repot the plants into a larger pot (diameter: 15 cm, height: 15 cm) with a new type of potting soil (for example: https://dcm-info.be/fr/pro, product 1002381) ( Figure 2–F). This type of soil should have a high-water holding capacity. Keep watering regularly, around two to three times a week. *Note: At this step, Teros 21 sensors were installed in the pots to monitor soil matric potential (Please refer to the Ψ _soil_ measurement protocol).*
e. After another two weeks, the plants are ready for the installation of PSY-1 leaf psychrometers, detailed in a subsequent section.

**Tomato**

a. Prepare a plastic tray (e.g. 38 × 25 × 6 *cm* [ *L*× *W*× *H*]) with drainage holes at the bottom, and fill it with a seedling & cuttings potting soil ( Figure 2H).
b. Transfer the germinated seeds ( Figure 2G) to the top of the soil (plant spacing ~3–4 cm, row spacing ~3–4 cm) and cover the seeds with ~0.5 cm of soil.
c. Water the soil until water drainage is seen from the bottom of the tray and cover the tray with glass to reduce the evaporation (remove it after one week) ( Figure 2I–J).
d. After two weeks, repot each plant into a larger pot (e.g. 6 × 6 × 6 *cm* ( *L*× *W*× *H*) with seedling & cuttings potting soil ( Figure 2K). Keep watering regularly, around two to three times a week.
e. After two weeks, repot the plants into a larger pot (diameter: 15 cm, height: 15 cm) with potting soil ( https://dcm-info.be/fr/pro, DCM, product 1002381) ( Figure 2L). Keep watering regularly, around two to three times a week. *Note: At this step, Teros 21 sensors were installed in the pots to monitor soil water conditions (Please refer to the Ψ _soil_ measurement protocol).*
f. After another two weeks, the plants are ready for the installation of PSY-1 leaf psychrometers, detailed in the next section.

### Soil and leaf water potential measurements

***1. Soil water potential (Ψ***_***soil***_ ***)***

Timing: 30~60 minutes for the installation. The duration of the measurements will depend on the experiments.

**Figure 3.**
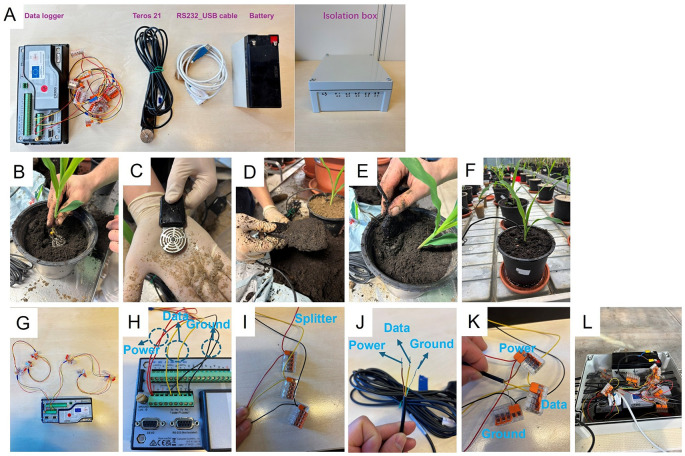
Installation of Teros21 in the pots. **A**, Materials for the installation of Teros 21. **B**- **F**, the installation of Teros 21 during repotting at section 2 (maize, steps d; tomato, steps e.) **G**- **L**, the connection between Teros 21 and data logger CR800.

**Figure 4.**
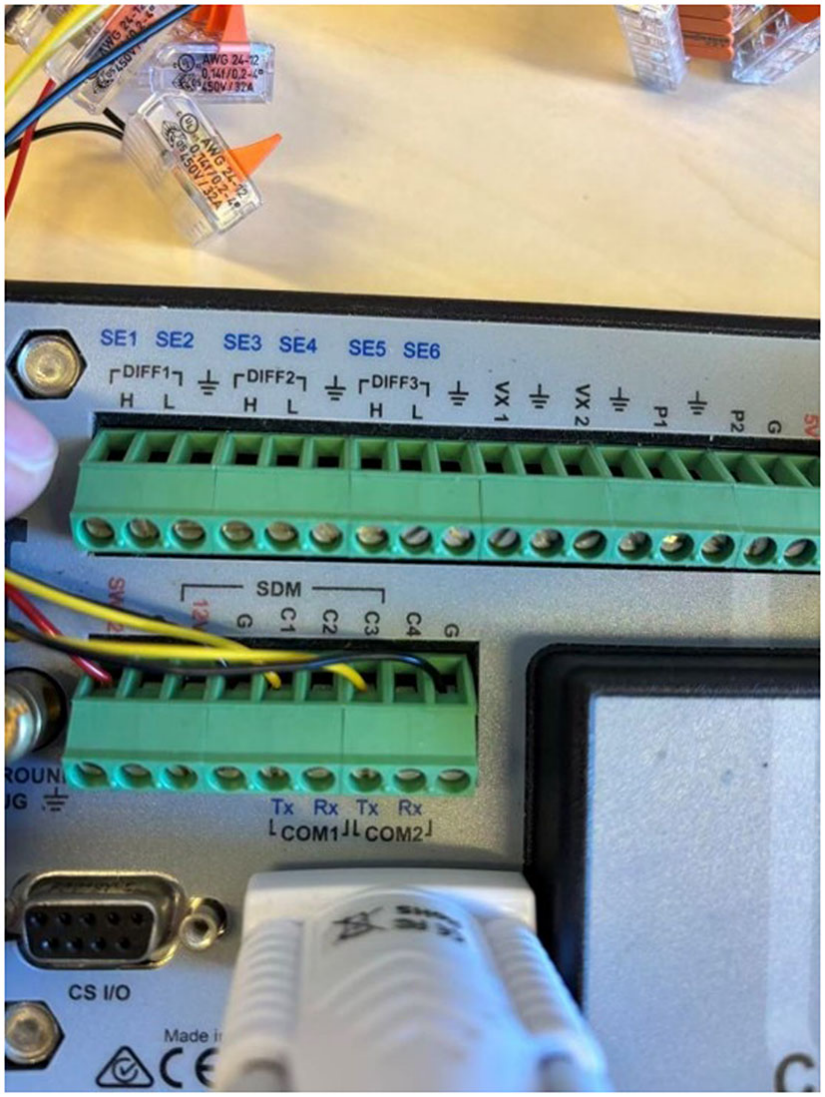
The data transporting ports between Teros21 and CR800.

**Figure 5.**
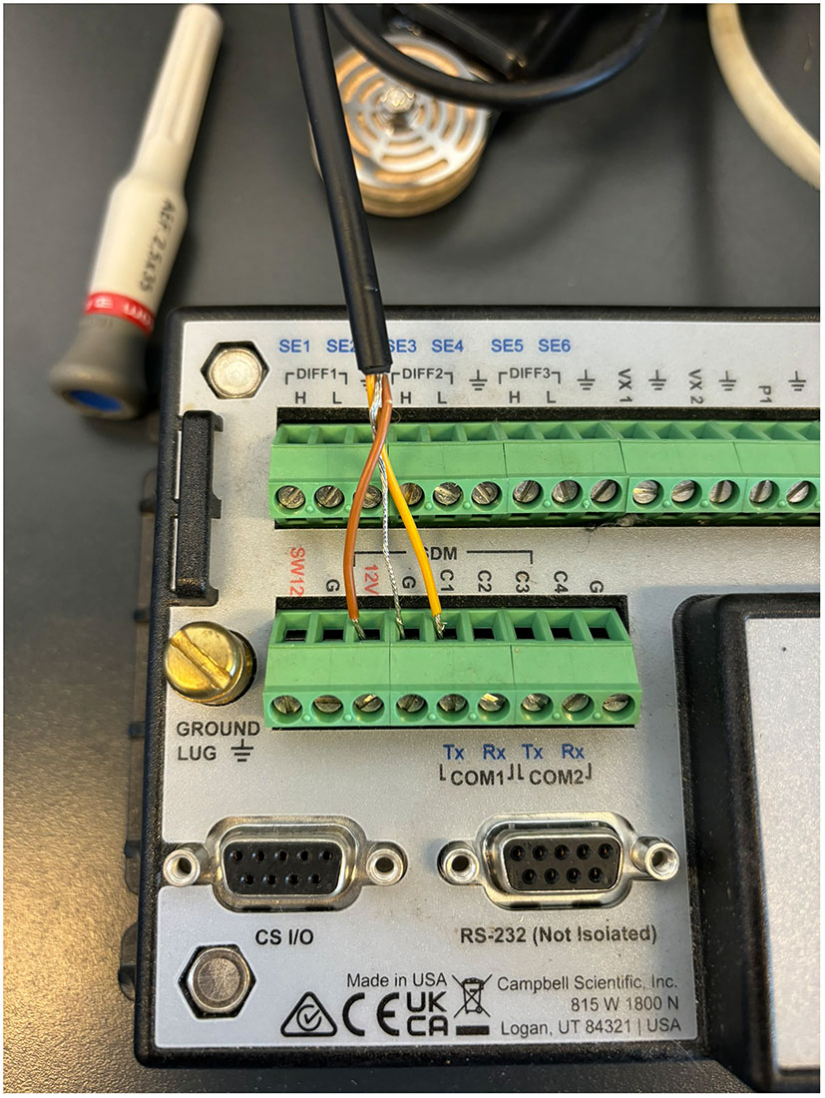
The connection of Teros21 and CR800 for checking and changing the address of the sensor.

Teros 21 is factory calibrated at 0 kPa, 0~−100 kPa (four points) and −100000 kPa. When soil matric potential < −100 kPa, TEROS 21 relies on the linear relationship between the logarithm of water content and the logarithm of water potential. No calibration will be needed prior to use. However, additional considering will be required to use Teros 21 when, 1) measuring in frozen soils; 2) measuring in high salinity; 3) temperature sensitivity at matric potential less than about −500 kPa.

a. Prepare the materials ( Figure 3A): Data logger (CR800, Campbell Scientific, Logan, Utah, United states), Teros 21 (Meter group, Washington, United States), RS232_USB cable, 12 V battery (Y7–12, Yuasa), isolation box.
b. Transfer the plant to the pot ( Figure 3B), as explained in the protocol for transplanting and repotting (maize, step “d”; tomato, step “e”).
c. To favor the contact between the sensor and the soil, cover Teros 21 with moist soil by gently pressing soil around the sensor ( Figure 3C-D).
d. Make a hole in the soil, put the sensor in the pot and fill the pot with soil ( Figure 3E-F). Keep the plants in the greenhouse for further growth before the installation of PSY1 (see the protocols for the installation of PSY1).
e. Connect the Teros 21 sensors with a CR800 in an isolation box ( Figure 3G-L). In this study, we used “C1” and “C3” ports from SDM channel ( Figure 4). In each port, it is possible to connect a maximum of 10 Teros 21 sensors with different addresses. Terminal blocks were used for multiple connections of Teros 21 to one port ( Figure 3I and K).
- Note: To check and change the address number for each sensor, connect an individual Teros 21 sensor to CR800 ( Figure 5), then follow the protocol from Campbell Scientific ( https://s.campbellsci.com/documents/us/technical-papers/sdi12-troubleshooting.pdf).
f. On a computer, write a program for the data logger with a programming tool (in this study, “CRBasic Editor” from Campbell Scientific was used, https://help.campbellsci.com/crbasic/cr1000x/). CR800 needs a program to send an operation to the Teros 21, read the SDI-12 bus communication protocol received by the Teros 21, and store the data. In this program, key information that will be sent and received by the SDI-12 signal, needs to be specified, i.e. (1) the address of each Teros 21; (2) the data transporting port (“C1” or “C3”); (3) the creation of dataset files to store the recorded soil matric potential and temperature, as well as the voltage of the battery for power supply; (4) the time interval for the data recording.
- An example of the program was deposited on Zenodo ( https://doi.org/10.5281/zenodo.17158115), with the document name of “Program-CR800”). Before starting, install the software of “Device Configuration Utility” and “PC400” from Campbell Scientific ( https://www.campbellsci.com/devconfig; https://www.campbellsci.com/pc400). “CRBasic Editor” is integrated inside PC400. For more details about the programming, please refer to https://www.campbellsci.eu/videos/datalogger-programming?slid=101#slide=99.
g. Flash the program to the CR800. Firstly, connect CR800 with power supply (either a 12 V battery or a 12 V adapter socket directly plugged from the wall (220–240 V)). Then, connect CR800 with computer by RS232_USB cable. In this study, “Device Configuration Utility” was used to interact with CR800, which includes sending the program, identifying Teros 21 address, and downloading the data.
- *Note: For more details of how to use “Device Configuration Utility”, please refer to the protocols from Campbell Scientific (* *https://www.campbellsci.com/devconfig* *).*
h. During the next day or after few hours of the installation, download the data from CR800 with “Device Configuration Utility” software to validate all the sensors working properly.
- *Note: For more details of how to use Teros 21, please also read the user manual from meter group (* *https://metergroup.com/products/teros-21/teros-21-support/* *).*

## **2. Leaf water potential (Ψ**_*leaf*_ **)**

Duration: ~5 minutes per installation. The duration of the measures will depend on the experiments. However,one installation could generate reliable data for one to two weeks for maize and tomato. After this period, the installed area of the leaf will be in senescence and non-functional for water transport, so a new installation will be required to change the position on the leaves. An additional explicative video for the psychrometer installation on leaves is available on Zenodo ( Degand *et al.*, 2025).

Note: PSY1 must be calibrated prior to first use or before installing in a new condition to guarantee accurate results. It is recommended to calibrate the instrument at or near the temperature at which it will normally be used. The calibration includes making measurements with calibration solutions of 0.1 M, 0.2 M, 0.3 M, 0.4 M,0.5 M, 1 M, 1.2 M NaCl on paper disk loaded to lid, to plot the data, generating a slope and an intercept that can be stored to calculate corrected water potentials in the ICT international software. After calibration, a good practice is to test that calibration with a 1 M NaCl solution that should give a value around −4.64 MPa after equilibration time (30 min to 2 hours). For other details of calibration, please check the manual from ICT international ( https://ictinternational.com/wp-content/uploads/2023/08/PSY1-Manual-DOC-00087v2.pdf).

**Figure 6.**
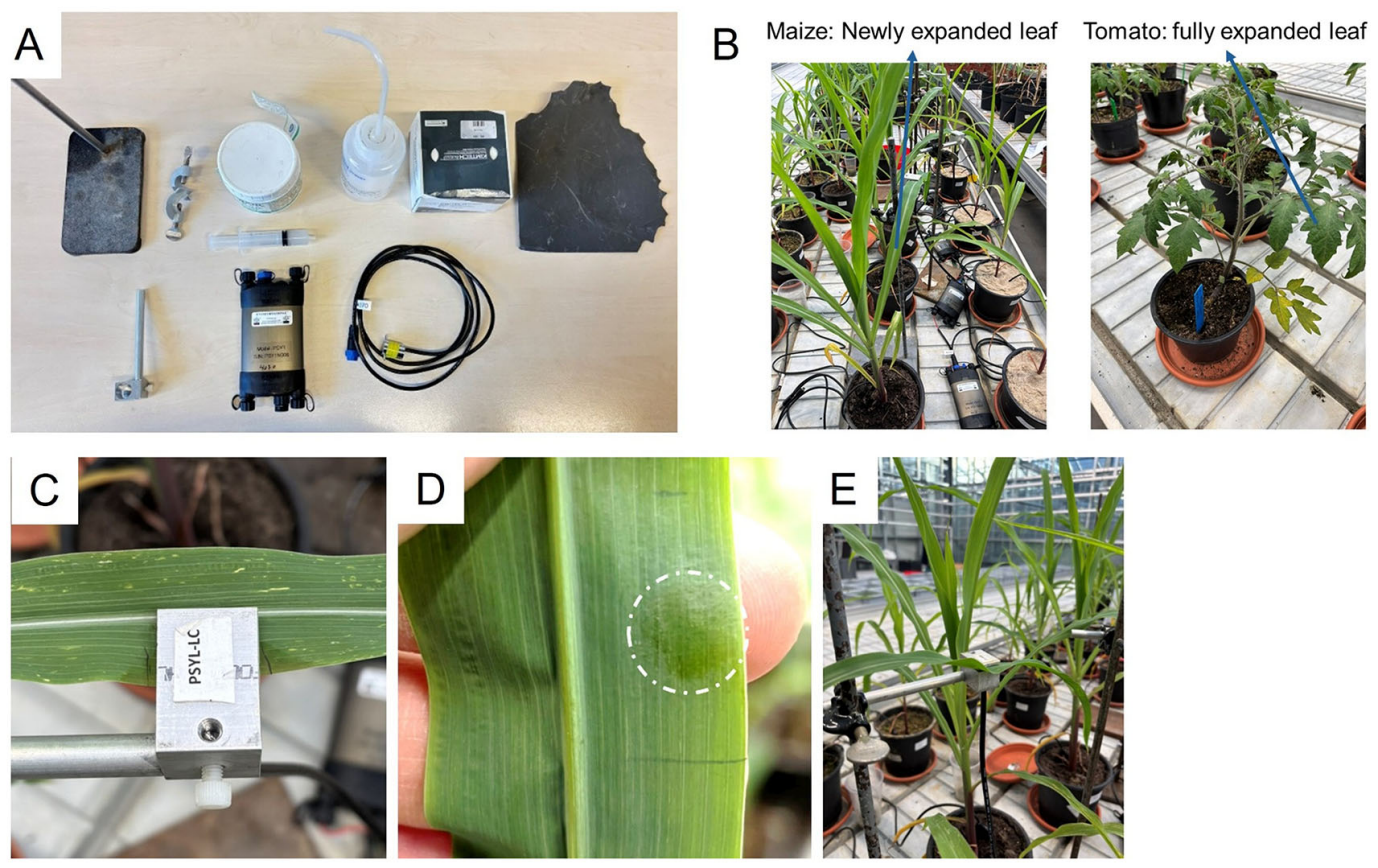
Installation of PSY1-Leaf on maize leaves. **A**, Materials needed for the installation. **B**, leaves selected for the measurement in maize and tomato. **C**, select the region for the installation. **D**, leaf after the abrasion with sandpaper. **E**, fix the head in the leaf clamp.

a. Prepare the materials ( Figure 6A): PSY1-leaf psychrometer (head and data logger, ICT international, Armidale, Australia), leaf clamps (PSY1-Leaf Psychrometer Clamps, ICT international, Armidale, Australia), retort stand, grease (Bardahl 1688 Silicone Grease, Bardahl, France), sandpaper (P400, grit size ~35 μm), demineralized water, precision wipes (Kimberly-Clark).
b. Choose newly expanded leaf for maize and fully expanded leaf for tomato (blue arrows in Figure 6B).
c. Set up the retort stand (with the leaf chamber attached) beside the selected leaf. The leaf should be able to rest freely inside the leaf chamber without being bent.
d. Put the selected leaf inside the leaf clamp and use a marker pen to mark the position on each side of the leaf ( Figure 6C).
e. Remove the leaf from the chamber, take a piece of sandpaper (~1 cm ^2^), lightly wet the sandpaper with a few drops of demineralized water for lubrication, and use the sandpaper to abrade the marked region on the abaxial side of the leaf ( Figure 6D). This abrasion will remove the cuticle on the surface of the leaf, in turn reducing cuticle resistance and improving water vapor diffusion in leaves. By removing cuticles, it will also reduce the equilibration time of leaf and psychrometer chamber. Ideally, the abrasion should just remove the cuticles without damaging the epidermal cells. It is recommended by the training from ICT international company to use a grit size of P400 for thick cuticles, such as citrus, a grit size of P600 for grasses, and a grit size of P800 for thin cuticles, such as beans.
f. Clean the abrasion region with demineralized water and dry it with tissue paper. Put the leaf back into the leaf clamp.
g. Take one PSY1-leaf psychrometer and put silicone grease around the edge of the cylindrical face. Spread the grease to have a ~0.5 mm layer of grease. This can be done either with a finger, or by placing the psychrometer lid back onto the chamber and turning slightly (remove lid after this action).
h. Put the psychrometer into the leaf clamp and fix it with the screw ( Figure 6E).
- Note: In this study, the psychrometer was used in the greenhouse, with no thermal insulation around the leaf clamp. In a separate experiment, it was found that the measurements of PSY1-leaf showed similar water potential values between insulated and non-insulated treatments in the greenhouse. However, limiting temperature gradients and maintaining constant instrument temperature are two highly desirable factors in the reliability of the measurements. In the field, insulating the chamber creates a thermal buffer zone to avoid rapid temperature change during the measurement. This insulation is not intended to prevent the diurnal temperature change but rather serves as a way to avoid condensation in the chamber, which would compromise the measurement. (For more details of the insulation, please refer to the manual from ICT international, https://ictinternational.com/wp-content/uploads/2023/08/PSY1-Manual-DOC-00087v2.pdf).
i. Connect the data logger of the psychrometer to a computer to configure the settings by following the instructions from ICT international. In this study, data was recorded every 15 minutes, with a cooling time of 10 seconds, waiting time of 6 seconds, warming duration of 0 seconds, waiting time before measurement of 15 seconds, and chamber heating of 0 seconds. However, these settings can be changed according to the experiment by following the instructions from ICT international (see “0” error section).
- Note: Installation of the ICT international software is required to connect psychrometers (Combined Instrument Software, ICT international, https://ictinternational.com/product/psy1-psychrometer-for-plant-water-potential/). Please follow the protocols given by ICT international to install the software and connect the PSY1-leaf sensors to a computer.
j. After allowing the leaf to equilibrate (typically between 30 minutes and 2 hours), the next step is to download and review the leaf water potential data. It is also good practice to check the data again about 24 hours after installation to confirm that a normal day–night pattern is observed. To retrieve the data, you can either connect the PSY1 leaf data logger directly to a computer using the dedicated software or remove the memory card from the logger and read it on a computer. If the installation was not done properly, the PSY1 may need to be reinstalled. Signs of improper installation often appear clearly in the data. For example, a typical dataset should show a diurnal pattern, with leaf water potential decreasing during the day as transpiration increases and recovering during the night. If this pattern is absent, or if the data consistently shows values close to “0”, it likely indicates an issue with the installation.

The “0” recording can be induced due to the abrasion technique, or due to a bending of the thermal couple, which might lead to a loss of contact with the leaf. In this case, check the thermocouple under a microscope and adjust the position if necessary, by following the protocols from ICT international ( https://www.youtube.com/watch?v=kNsZnSYDBPE&list=PL4F3FBBF0881B16D7&index=16).

Lastly, a common issue when working with psychrometers is condensation. If the sample tissue is cooler than the chamber, water may condense directly on the sample, where it can be absorbed or redistributed. On the other hand, if the chamber is cooler than the sample, condensation may form on the chamber walls, introducing uncertainty and potential errors in the measurements. This problem is often noticeable in the data when there are strong temperature gradients (dT). In most cases, condensation issues can be resolved by cleaning the system and reinstalling the components properly. However, if the problem persists, it may be necessary to adjust certain parameters, such as the cooling time or chamber heating settings. For more details on these adjustments, refer to Chapter 6 (“Settings and Measurement Options”) of the PSY1 Manual DOC-00087v2.If none of these solutions resolve the issue—particularly when measurements remain close to 0 MPa—it is important to recognize a fundamental limitation of the psychrometric method. Near the wet end of the spectrum (around 0 MPa), the energy required to convert water from liquid to vapor is extremely low, which reduces measurement sensitivity and accuracy. This situation is uncommon in potted plants but can occur more frequently in hydroponic systems.

Download and check the data regularly (once a day). This regular check will help in tracking the data, and in identifying any issues (as mentioned above) as early as possible.

As a complement to leaf water potential, plant transpiration can be monitored using weighing scales. In this study, whole-plant transpiration was monitored continuously by recording pot weight over the same duration of the psychrometers. The pots were placed on a scale (A&D, EK-15KL, Japan), and the weight was recorded every 15 minutes using the RsWeight program from the Windows Communication Tools (WinCT) package. This software, developed by A&D, collected data via a StarTech USB 16- or 8-port adapter connected to the scale through an RS232 cable. To minimize soil evaporation, the pots were covered with aluminum foil.

Additionally, destructive measurements of Ψ *_leaf_* can be taken using a Scholander pressure bomb to compare with the data measured by TCP. However, it is still unclear whether the Scholander pressure bomb measures both Ψ _*p*_ and Ψ _*o*_ or just the Ψ _*p*_ of leaves, while TCP measures both Ψ _*p*_ and Ψ _*o*_.

## Data analysis

Timing: ~10 minutes.

**Figure 7.**
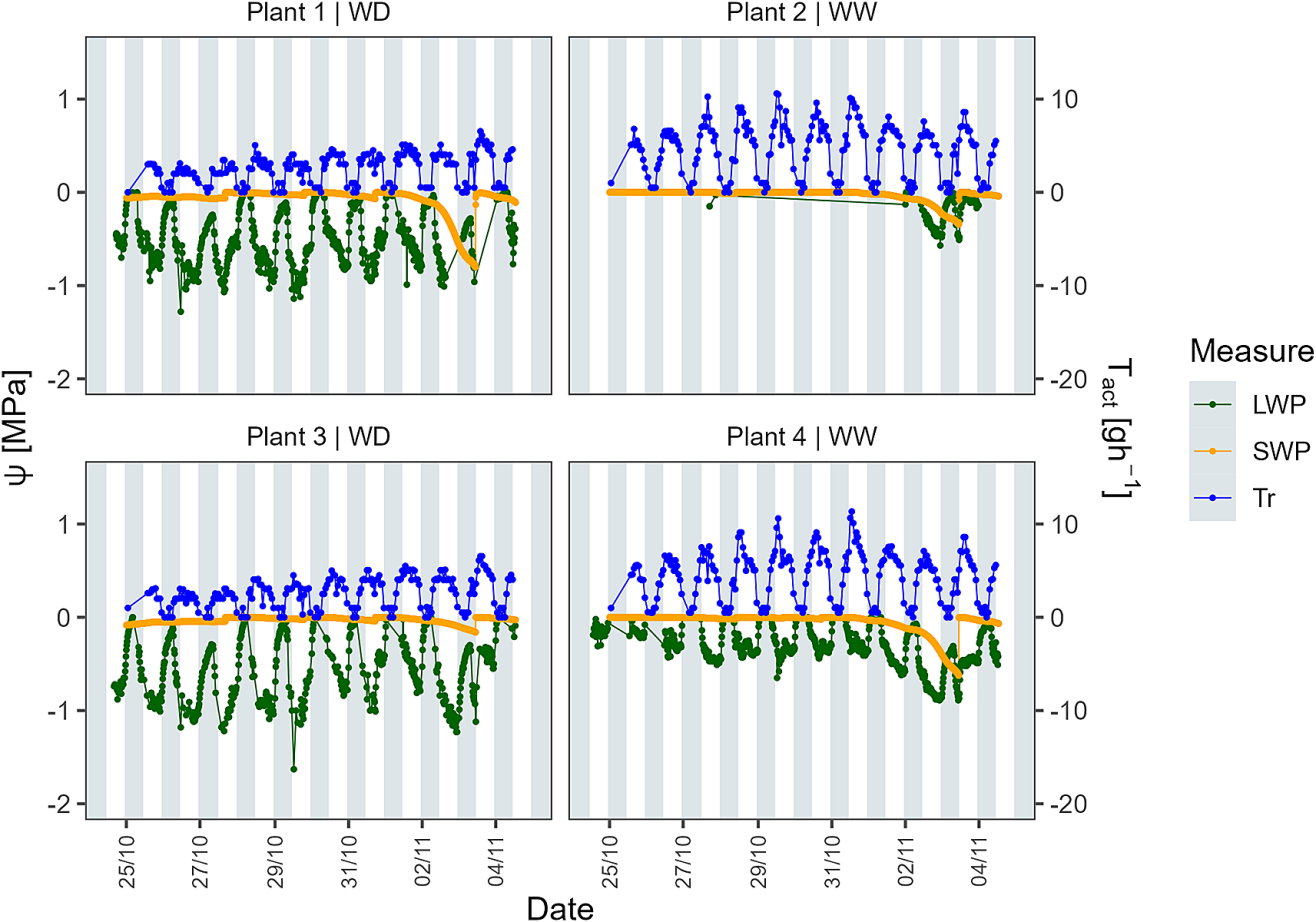
Example of compiled and cleaned data from the R notebook.

We made an R quarto document as well as custom functions to load raw measurements files, clean the data files, and make graphs. This R quarto document is available on Zenodo ( D’Agostino, 2026). The folder contains a user-friendly notebook to load files in R, custom functions to extract and compile data, and a simple dataset containing data about 4 plants. Figure 7 shows compiled and cleaned data from the example dataset.

***1) Code setup***

Download the repository ( D’Agostino, 2026). Extract the content to a local disk. Launch Rstudio. Open the file *“Data_Analysis_Main.qmd*” located in the repository folder. Ensure all required libraries are installed.

***2) Data collection***

Collect data from both Teros 21 and PSY1-leaf by connecting the data loggers to a computer with the relevant software mentioned previously. PSY1-leaf will generate a.csv format file and store all the data inside, while CR800 generates a DAT format file. In both cases, copy each individual file to *data/raw/LWP/*folder. Make sure that each file is named as the ID of the sensor.

***3) Encode your metadata***

Create a metadata file that contains identifiers for plants and sensors, similar to *Plants_ID.csv* in *data/clean/*folder. Make sure that each sensor ID corresponds to the right Plant_ID.

***4) Run the code to extract and clean leaf water potential data***

The code automatically extracts data from each individual LWP file and integrates it into one unique table that contains LWP data per date and per Plant_ID. The table is saved in the *data/raw/*folder. Statistical manipulations or data corrections can then be applied as deemed appropriate. A cleaning algorithm has been included in the code that filters data based on: the number of “0” and *“NAN”* values; incorrect day-night cycle amplitude; the global slope, and the global level (more details are provided in the notebook).

A web version of the notebook is available here: https://theplantwaterpump.github.io/TS_Protocol/

***5) Run the code to extract soil water potential and transpiration data***

A similar process to the leaf water potential is done for soil water potential (SWP) data and weight data within the code. Follow instructions set in the notebook for further detail for these two data types. Additionally, the code includes the computation of transpiration rate, and allows all variables to be plotted together ( Figure 7).

Because this protocol paper is intended to demonstrate PSY1 operation and interpretation, the example dataset is illustrative and not designed for rigorous statistical treatment; users should include sufficient biological replication, typically at least 3–5 replicates where possible depending on the experimental objective and biological variability, together with calibration checks and uncertainty analysis.

## Troubleshooting

### Heterogeneity of water distribution in the soil

As the soil dries out, there will be heterogeneity of water distribution in the soil. To have more representative Ψ *_soil_* measurements in the pot, we recommend to:

(1) Install Teros 21 at different depths and/or the same depth with different horizonal positions.
(2) Avoid watering in one location and instead try to spray the water uniformly over the whole surface of the top layer of the soil. If a large amount of water is required to be given to the plant, try to split the amount into several portions and give it to the plant during the day.

### Charging issue for the psychrometer data recording

**Figure 8.**
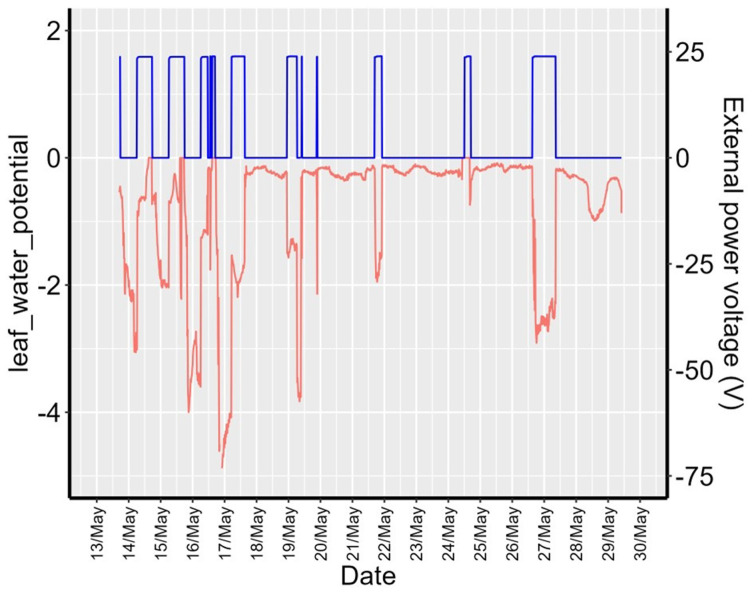
Example of the charging effects on the data recordings.

During this study, difficulties with the psychrometer data were encountered due to the impact of the data logger’s charging process. Fluctuations in the PSY1 data were exhibited when the charging process was initiated or terminated (as shown in Figure 8). This occurred particularly when the PSY1 data loggers were connected to a power socket simultaneously used by other devices, such as weighing scales. To avoid this issue, an external 12 V battery was used to charge the data loggers.

### Data cleaning with an algorithm

**Figure 9.**
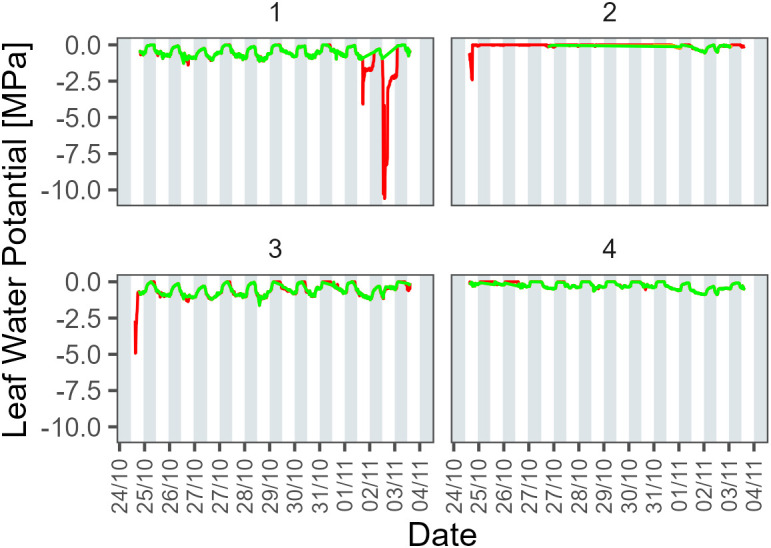
Example of data cleaning using the algorithm. Green is kept data and red is discarded data.

The initial dataset from the psychrometer measurements may contain several outliers, missing values (skipped due to low battery), incorrect entries, and aberrant patterns. To resolve this, a data cleaning algorithm was implemented. This algorithm was designed to remove any values that were missing, zero, or otherwise aberrant. It was also programmed to identify and remove inverted day-night cycle patterns, as well as values that were statistically insignificant. Figure 9 shows applications of data cleaning on the example dataset. For more details, please check codes that have been deposited on Zenodo ( https://doi.org/10.5281/zenodo.20080750, D’Agostino, 2026).

## Conclusion

In summary, the present protocol is not confined to the descriptive monitoring of Ψ *_soil_* and Ψ *_leaf_*; it also furnishes an experimental framework to relate continuous water-potential measurements to plant hydraulic properties. As previously mentioned, radial water flow can be expressed as a function of the driving force in Ψ *_tot_* and the corresponding hydraulic conductivity. Accordingly, when TEROS 21 measurements of soil matric potential are interpreted in conjunction with PSY1-derived Ψ *_leaf_* and an independent estimate of water flux, such as whole-plant transpiration obtained gravimetrically, these time series can be used to assess changes in apparent root or whole-plant hydraulic conductance during the processes of drying and rewetting. It is imperative to note that such an interpretation necessitates explicit assumptions, including hydraulic equilibration at the measurement interface, limited temperature artefacts, and a physically consistent comparison between the water-potential components under consideration. It has been demonstrated that, within the established limits, the combined measurements are not only useful for tracking plant water status over time, but also for identifying phases of hydraulic limitation, soil-root disconnection, and recovery. Consequently, this linkage of the protocol to mechanistic analyses of water transport in the SPAC is more direct.

## Ethics and consent

Ethical approval and consent were not required.

## Data availability

The datasets and codes to analyze the data have been deposited on Zenodo ( https://doi.org/10.5281/zenodo.20080750, D’Agostino (2026)).

Data are available under the terms of the Creative Commons Zero v1.0 Universal.

An additional explicative video for the psychrometer installation on leaves is available on Zenodo ( https://doi.org/10.5281/zenodo.17510720, Degand *et al.* (2025)).

The author(s) declare that this video is released under the Creative Commons CC0 1.0 Universal Public Domain Dedication. This means the video is free of all copyright restrictions and may be copied, modified, distributed, and used without permission, including for commercial purposes.

Data are available under the terms of the Creative Commons Attribution 4.0 International.
