# Supplementary material for: Protocols for in situ continuous monitoring of water relations/potential in soil and leaf
Source: Open Res Eur. 2026 Sep 28;5:363. Originally published 2025 Nov 27. [Version 3] doi: 10.12688/openreseurope.21382.3 (PMC13287980; doi:10.12688/openreseurope.21382.3)
Supplement: Peer review history

# Reviewer report: Reviewer response for version 2

**DOI:** 10.21956/openreseurope.25594.r76009

**Published:** 2026-07-01

**Authors:** Tina Koehler

**Affiliations:** 1 Technical University of Munich, Freising, Germany

General comments:

 The manuscript entitled “Protocols for in situ continuous monitoring of water relations/potential in soil and leaf” by Ding and colleagues is timely (i.e., it relates well to recently published work on the topic, e.g., in reference), and important in terms of offering a standardized protocol for applying in-situ measurements of soil- and leaf water potential, thereby enhancing the comparability across experiments conducted by different groups. The scripts they share on publicly available repositories are especially appreciated. I believe the present manuscript can contribute meaningfully to the community conducting those measurements, and, more importantly, help bridge the gap to communities that are not yet employing these measurements. However, I have some suggestions for refinements, which are specified below.

- Avoid jargon like “Light was …” when you mean “ *light intensity was* …”, or “To retrieve the data, *you can* either…”
- Unify use of abbreviations (e.g., sometimes you use LWP, sometimes “Leaf water potential”, and sometimes Ψ _leaf_, in the text as well as in the figure axis titles and legends)
- Unify spelling (e.g., sometimes you use Teros21 and sometimes TEROS21)
- Use consistent variables, titles, and colors across plots (e.g., no proper left y-axis title nor units in Fig. 8, and no meaningful subplot titles in Fig. 7)

P3:

- “It will be assumed herein that frictions along xylem vessels are negligible for the small plants of this study, and xylem water potential in the root and leaf are equivalent” - regarding this statement, it would be good to add a “Note that..” and mention some of the studies addressing, e.g., xylem-internal friction.
- When referring to methods for quantifying leaf water potential, it would be good to refer to the article in the reference section.
- Consider referring to key papers contributing to the debate around whether the Scholander pressure chamber measures total leaf water potential or merely the pressure potential.

P4:

- Helpful introduction to which sensors and methods are commonly used to quantify leaf and soil water potential. I would add a sentence on why exactly TEROS21 and PSY1 psychrometers were chosen amongst all of the introduced ones for *this* study.
- TEROS21 does not work well in the wet range. METER even specifies –8 to –100.000 kPa.
- “Despite the fact that PSY1 measurements are affected by the soil matric water potential reported by TEROS 21, a direct comparison between the two is not recommended” - What do you mean by *a direct comparison*? Of course, there is a temporal and magnitude offset between leaf water potential and soil water potential, and diurnal patterns of leaf water potential do not necessarily translate into (measurable) diurnal fluctuations in soil water potential (especially in wet soil). However, interpreting leaf water potential without knowing the water status of the soil and vice versa will lead to invalid comparisons. It would help if this statement could be made clearer.

P5:

- In 1c., you refer to “the Sandwich technique in d.”, and I am not sure which d. you mean.
- “Artificial light [intensity] was 160-170 µmol m ^-2^ s ^-1^” - that seems quite low. I would report the actual light intensity the plants experienced.

P6:

- Maize b.: How deep to plant the seeds if they are not pre-germinated?
- Maize c.: Why irrigation with rainwater, specifically? Do you imply de-mineralized water?
- Maize d.: Why plant maize in a new type of potting soil upon transplantation?
- Maize d.: Instead of saying that the soil should be watered “regularly”, which is very vague, it would be more helpful (and in line with the paper) to indicate a specific soil water potential level/ range to aim for and possibly suggest a protocol for determining how much water to add (weight or volume).

P7:

- Tomato e.: I would avoid going into the details of recommending how often to repot. For example, researchers interested in root architecture might not want to repeatedly disturb root development. I think this aspect of the plant establishment and growth will be rather context specific.
- Soil water potential a.: The METER ZL6 logger could also be used instead of programming a Campbell logger. Is there any specific reason why you decided on the other way?
- Soil water potential d.: The installation of the TEROS21 could be specified in greater detail, as this is crucial for the quality of the data. For example, the sensors should be as far away from the pot walls as possible and, ideally, installed horizontally rather than vertically.

P8:

- Soil water potential e.: Are “Terminal blocks” the same as “splitters” (in Fig. 3I)? If yes, use same terminology.
- Soil water potential f.: Thank you for depositing the file for programming the Campbell logger on Zenodo, I am sure this will be very helpful for the community.

P9:

- Soil water potential h.: Fix grammar: “to validate [if] all the sensors [are] working properly”
- Leaf water potential: Does water transport functionality decline gradually as the leaf on which the psychrometer is installed senesces? If yes, from when on do the data become unreliable?
- Leaf water potential b.: When you say “newly expanded leaf for maize and fully expanded leaf for tomato”, do you mean “youngest fully developed leaf for maize”? It should already be fully expanded, also for maize, right?

P11:

- The comment on the importance of buffering psychrometers against temperature changes is really important here. Could you add a plot of temperature (and possibly relative humidity) fluctuations over the experimental period in the greenhouse here? Typically, it can get quite hot inside greenhouses. Hence, I am surprised that you do not see a signal of this in your psychrometer data.
- Specify the resolution of the balance. This matters for resolving diurnal plant water-use patterns in small, potted plants.

P14:

- Not sure if changes in whole-plant hydraulic conductance *during* the process of drying and rewetting can be inferred from time series of transpiration rate, soil- and leaf water potential, as Darcy’s law, that is typically being used for that, applies under steady-state conditions only…

Is the study design appropriate for the research question?

Yes

Is the rationale for, and objectives of, the study clearly described?

Yes

Are sufficient details of the methods provided to allow replication by others?

Partly

Are the datasets clearly presented in a useable and accessible format?

Partly

Reviewer Expertise:

soil physics, soil-plant hydraulics, plant/ crop physiology

I confirm that I have read this submission and believe that I have an appropriate level of expertise to confirm that it is of an acceptable scientific standard, however I have significant reservations, as outlined above.

## References

[1] : Continuous monitoring of plant water potential: sensor‐based approaches and best practices. *New Phytologist* .2026;251(1) : 10.1111/nph.71194569-588 10.1111/nph.71194 42087530

# Response

**Published:** 2026-09-07

**Authors:** Lei Ding

**Affiliations:** Earth and life institute, Universite catholique de Louvain, Louvain-la-Neuve, Walloon Region, Belgium

Reviewer 4 General comments:

 The manuscript entitled “Protocols for in situ continuous monitoring of water relations/potential in soil and leaf” by Ding and colleagues is timely (i.e., it relates well to recently published work on the topic, e.g., in reference), and important in terms of offering a standardized protocol for applying in-situ measurements of soil- and leaf water potential, thereby enhancing the comparability across experiments conducted by different groups. The scripts they share on publicly available repositories are especially appreciated. I believe the present manuscript can contribute meaningfully to the community conducting those measurements, and, more importantly, help bridge the gap to communities that are not yet employing these measurements. However, I have some suggestions for refinements, which are specified below.

- Avoid jargon like “Light was …” when you mean “ *light intensity was* …”, or “To retrieve the data, *you can* either…”

Answer: Thanks for this suggestion! We revised the text.

- Unify use of abbreviations (e.g., sometimes you use LWP, sometimes “Leaf water potential”, and sometimes Ψ _leaf_, in the text as well as in the figure axis titles and legends)

Answer: We used Ψ _leaf  _and Ψ _soil_ throughout the text and figures in the revised manuscript.

- Unify spelling (e.g., sometimes you use Teros21 and sometimes TEROS21)

Answer: Teros 21 was used in the revised manuscript.

- Use consistent variables, titles, and colors across plots (e.g., no proper left y-axis title nor units in Fig. 8, and no meaningful subplot titles in Fig. 7)

Answer: We have revised Figure 7 and 8, please check! P3:

- “It will be assumed herein that frictions along xylem vessels are negligible for the small plants of this study, and xylem water potential in the root and leaf are equivalent” - regarding this statement, it would be good to add a “Note that..” and mention some of the studies addressing, e.g., xylem-internal friction.

Answer: We thank the reviewer for this good suggestion. We cited Venturas et al. (2017). Reference Venturas MD, Sperry JS, Hacke UG: Plant xylem hydraulics: What we understand, current research, and future challenges. Journal of Integrative Plant Biology. 2017;59 (6): 356–389 10.1111/jipb.12534

- Consider referring to key papers contributing to the debate around whether the Scholander pressure chamber measures total leaf water potential or merely the pressure potential.

Answer: Thank you for this suggestion. We have included several key references discussing the ongoing debate regarding whether the Scholander pressure chamber measures total leaf water potential (Ψ_tot) or primarily xylem pressure potential (Ψ_p). The corresponding text has been revised as follows: Concurrently, the Scholander pressure chamber proposed a direct but destructive measurement of (Ψ *_leaf_*)  (Scholander *et al.*, 1965). Whether this technique measures Ψ *_tot_* or Ψ *_p_* remains a matter of debate (Boyer, 1969; Rodríguez-Domínguez et al., 2022). The method assumes that equilibrium is reached between the applied balancing pressure and the negative water potential in the leaf xylem, while the osmotic potential of the xylem/apoplastic sap is negligible (Boyer, 1969). However, this assumption may not always hold, as differences among measurement techniques and the presence of non-negligible xylem osmotic potential under certain physiological or environmental conditions can influence the interpretation of pressure chamber measurements (Rodríguez-Domínguez et al., 2022; Enns et al., 2000; Westhoff et al., 2008).   P4:

- Helpful introduction to which sensors and methods are commonly used to quantify leaf and soil water potential. I would add a sentence on why exactly TEROS21 and PSY1 psychrometers were chosen amongst all of the introduced ones for *this* study.

Answer: We explained why we selected Teros 21 and PSY1 in this study in the revised manuscript, please check.  

- TEROS21 does not work well in the wet range. METER even specifies –8 to –100.000 kPa.

Answer: According to the user manual, Teros 21 detection range is 0 to –100.000 kPa.  

- “Despite the fact that PSY1 measurements are affected by the soil matric water potential reported by TEROS 21, a direct comparison between the two is not recommended” - What do you mean by *a direct comparison*? Of course, there is a temporal and magnitude offset between leaf water potential and soil water potential, and diurnal patterns of leaf water potential do not necessarily translate into (measurable) diurnal fluctuations in soil water potential (especially in wet soil). However, interpreting leaf water potential without knowing the water status of the soil and vice versa will lead to invalid comparisons. It would help if this statement could be made clearer.

Answer: Thank you for this helpful comment. We agree that leaf water potential and soil water potential provide complementary information and should be interpreted together to understand the soil–plant water continuum. Our original wording was unclear. By “direct comparison”, we intended to indicate that the absolute values of leaf water potential and soil matric potential should not be directly equated because they differ in both temporal dynamics and magnitude due to hydraulic resistance, plant water storage, and environmental conditions. We have revised the text accordingly to clarify this point.   P5:

- In 1c., you refer to “the Sandwich technique in d.”, and I am not sure which d. you mean.

Answer: Sorry for this confusing, we removed the text of “in d.”  

- “Artificial light [intensity] was 160-170 µmol m ^-2^ s ^-1^” - that seems quite low. I would report the actual light intensity the plants experienced.

Answer: In the original text, we mentioned “the artificial light intensity was 160~170 μmol.m ^−2^.s ^−1^ at table level”. However, the plants were grown in a glasshouse and there was sunlight as well. We did not record the actual light intensity during the period of this protocol study.   P6:

- Maize b.: How deep to plant the seeds if they are not pre-germinated?

Answer: We have now added the following clarification to the revised manuscript: “In the case of direct sowing, seeds are placed at a depth of approximately 1 cm in the soil.”  

- Maize c.: Why irrigation with rainwater, specifically? Do you imply de-mineralized water?

Answer: Sorry for this unclear! In our greenhouse facility, rainwater is collected and routinely used for plant irrigation. In this study, the maize plants were therefore irrigated with collected rainwater rather than demineralized water. We do not intend to imply that rainwater is equivalent to demineralized water, as it may contain small amounts of dissolved minerals and nutrients. We have revised the manuscript to clarify this point.  

- Maize d.: Why plant maize in a new type of potting soil upon transplantation?

Answer: In a, we mentioned that “This experiment used seedling & cuttings potting soil ( https://dcm-info.be/fr/pro, DCM, product 1004525), ensuring good water distribution, drainage and allowing the soil to retain its aerated structure”. In d, we mentioned that “…with a new type of potting soil (for example: https://dcm-info.be/fr/pro, product 1002381) ( Figure 2–F). This type of soil should have a high-water holding capacity. Keep watering regularly, around two to three times a week.”  

- Maize d.: Instead of saying that the soil should be watered “regularly”, which is very vague, it would be more helpful (and in line with the paper) to indicate a specific soil water potential level/ range to aim for and possibly suggest a protocol for determining how much water to add (weight or volume).

Answer: We thank the reviewer for this valuable suggestion. We agree that specifying a target soil water status is more informative than using the vague term “regularly.” However, we prefer not to prescribe a fixed irrigation volume because plant water requirements vary with environmental conditions, including radiation, temperature, and humidity, as well as with plant size and developmental stage. In our experiments, irrigation was adjusted according to soil water potential measured with the TEROS 21 sensor, together with transpiration-driven water loss. For well-watered conditions, pots were irrigated until drainage occurred from the bottom of the pot. For drought treatments, irrigation was withheld until the desired soil water potential was reached when TEROS 21 was installed and was then resumed according to the experimental design. We have revised the manuscript to clarify this procedure.   P7:

- Tomato e.: I would avoid going into the details of recommending how often to repot. For example, researchers interested in root architecture might not want to repeatedly disturb root development. I think this aspect of the plant establishment and growth will be rather context specific.

Answer: Thank you for this helpful suggestion. We agree that the timing and frequency of repotting are highly dependent on the experimental objectives. Our recommendation was intended for studies focusing on continuous in situ monitoring of soil and leaf water potential rather than experiments investigating root architecture or other root-related traits, where minimizing root disturbance is important. We have therefore revised the manuscript to remove the prescriptive recommendation on repotting frequency and instead state that repotting should be adjusted according to the specific experimental objectives.  

- Soil water potential a.: The METER ZL6 logger could also be used instead of programming a Campbell logger. Is there any specific reason why you decided on the other way?

Answer: Yes, there are several options for communicating with and recording data from the TEROS 21 sensor, including the METER ZL6 data logger. We used a Campbell Scientific data logger in this study because it was already available in our experimental setup and could be readily integrated with our other measurements. There is no specific methodological requirement to use a Campbell logger, and the ZL6 or other compatible logging systems can also be used.  

- Soil water potential d.: The installation of the TEROS21 could be specified in greater detail, as this is crucial for the quality of the data. For example, the sensors should be as far away from the pot walls as possible and, ideally, installed horizontally rather than vertically.

Answer: Thank you for this helpful suggestion! We revised the text in the revised manuscript.   P8:

- Soil water potential e.: Are “Terminal blocks” the same as “splitters” (in Fig. 3I)? If yes, use same terminology.

Answer: Yes, they are the same. We changed “Terminal blocks” to “splitters”.

- Soil water potential f.: Thank you for depositing the file for programming the Campbell logger on Zenodo, I am sure this will be very helpful for the community.

Answer: Thanks! P9:

- Soil water potential h.: Fix grammar: “to validate [if] all the sensors [are] working properly”

Answer: Done.  

- Leaf water potential: Does water transport functionality decline gradually as the leaf on which the psychrometer is installed senesces? If yes, from when on do the data become unreliable?

Answer: Water transport functionality does not decline gradually. We mentioned that “However, one installation could generate reliable data for one to two weeks for maize and tomato. After this period, the installed area of the leaf will be in senescence and non-functional for water transport, so a new installation will be required to change the position on the leaves.” We will need to check the data regularly to validate if the data becomes unreliable.

- Leaf water potential b.: When you say “newly expanded leaf for maize and fully expanded leaf for tomato”, do you mean “youngest fully developed leaf for maize”? It should already be fully expanded, also for maize, right?

Answer: Yes, we mean the youngest fully expanded leaf in maize. Leaves that are still elongating or expanding are not suitable for measurement because ongoing leaf growth can disturb sensor contact and may also cause mechanical damage to the leaf during the measurement period.   P11:

- The comment on the importance of buffering psychrometers against temperature changes is really important here. Could you add a plot of temperature (and possibly relative humidity) fluctuations over the experimental period in the greenhouse here? Typically, it can get quite hot inside greenhouses. Hence, I am surprised that you do not see a signal of this in your psychrometer data.

Answer: We thank the reviewer for this helpful suggestion. We have now added the PSY1 chamber temperature recorded over the experimental period together with the corresponding leaf water potential measurements (Figure 1). As expected under greenhouse conditions, chamber temperature showed a clear diurnal pattern. It is important to distinguish these variations in absolute chamber temperature from temperature gradients within the psychrometer system. The PSY1 can operate across changes in ambient temperature provided that the leaf, chamber, and sensor remain sufficiently close to thermal equilibrium. The leaf water potential measurements also showed diurnal dynamics. These variations may partly reflect the physiological response of the plants to changing daytime environmental conditions, including temperature and associated changes in transpiration demand, rather than a direct temperature artefact of the psychrometer. As mentioned in the manuscript, abnormal temperature gradients (dT), rather than chamber temperature alone, were used as an indicator of potentially unreliable PSY1 measurements.  

https://openreseurope-files.f1000.com/linked/280181.Lei_Ding.png

 Figure 1 Continuous recording of leaf water potential (Ψleaf; left y-axis, lower/thicker curve in each panel) and PSY1 chamber temperature (right y-axis, upper/thinner curve) for four plants during the experimental period.

- Specify the resolution of the balance. This matters for resolving diurnal plant water-use patterns in small, potted plants.

Answer: In this study, the resolution of the balance is 0.1 g. We mentioned this in the revised manuscript.   P14:

- Not sure if changes in whole-plant hydraulic conductance *during* the process of drying and rewetting can be inferred from time series of transpiration rate, soil- and leaf water potential, as Darcy’s law, that is typically being used for that, applies under steady-state conditions only…

Answer: We agree with the reviewer that changes in whole-plant hydraulic conductance during the process of drying and rewetting may not be inferred from time series of transpiration rate, soil- and leaf water potential, as Darcy’s law, that is typically being used for that, applies under steady-state conditions. In the revised manuscript, we mentioned that the assessment should be working under near-steady-state conditions and with appropriate assumptions.

# Reviewer report: Reviewer response for version 2

**DOI:** 10.21956/openreseurope.25594.r76008

**Published:** 2026-06-22

**Authors:** Simon Kraatz

**Affiliations:** 1 USDA ARS Hydrology and Remote Sensing Laboratory, Beltsville, Maryland, USA

This work is to provide measurement protocols for measuring water potential in tomato and maize starting at germination. The protocols are tailored to the PSY1 psychrometer and the TEROS21 matric potential sensor, but the concepts and basic steps provided likely also are more generally applicable. 

 The authors also provide an installation video of the leaf psychrometer, and an R code for data processing. 

 Given the level of detail (plus the installation video and code) this protocol should now be reproducible by others. 

 I agree with the previous two reviewers on the issue about errors inherent to psychrometry, but they have since been added to the protocol. 

 However, a key item still missing, the expected level of uncertainty for applying this protocol under standard conditions. There is instrument error but also between ideal conditions and in the theory, and how temperature gradient / non-equilibrium conditions etc. propagate. 

 There should be more detail on the 'otherwise aberrant' part in the data cleaning, and rationale should be given for removing inverted day night cycle patterns (why does this occur?).

 The manuscript text did define the abbreviations for the legend items shown in Figure 7 for SWP but not for Tr or LWP. Figure 7 caption could have more details.

 It is important to caution about the use of "Measure" in the caption, because the potentials are not direct measurements, they are calculated from e.g. vapor pressure measurements using the presented theory. All three quantities should have some error bar or uncertainty estimate due to instrument error & theory error propagation.

 Figure 8 should have a legend for the blue and red line, and the caption could have more information on what issue is presented in the Figure.

Is the study design appropriate for the research question?

Partly

Is the rationale for, and objectives of, the study clearly described?

Yes

Are sufficient details of the methods provided to allow replication by others?

Yes

Are the datasets clearly presented in a useable and accessible format?

Yes

Reviewer Expertise:

Sensing of plant and soil properties

I confirm that I have read this submission and believe that I have an appropriate level of expertise to confirm that it is of an acceptable scientific standard, however I have significant reservations, as outlined above.

# Response

**Published:** 2026-09-07

**Authors:** Lei Ding

**Affiliations:** Earth and life institute, Universite catholique de Louvain, Louvain-la-Neuve, Walloon Region, Belgium

Reviewer 3 This work is to provide measurement protocols for measuring water potential in tomato and maize starting at germination. The protocols are tailored to the PSY1 psychrometer and the TEROS21 matric potential sensor, but the concepts and basic steps provided likely also are more generally applicable. The authors also provide an installation video of the leaf psychrometer, and an R code for data processing. Given the level of detail (plus the installation video and code) this protocol should now be reproducible by others. I agree with the previous two reviewers on the issue about errors inherent to psychrometry, but they have since been added to the protocol. However, a key item still missing, the expected level of uncertainty for applying this protocol under standard conditions. There is instrument error but also between ideal conditions and in the theory, and how temperature gradient / non-equilibrium conditions etc. propagate. 

 Answer: We thank the reviewer for this important comment. We agree that understanding the expected level of uncertainty is important for the appropriate application and interpretation of this protocol. Based on our experience, high-quality and reproducible datasets can be obtained under standard experimental conditions, including both greenhouse and growth-chamber experiments, when the sensors are properly calibrated and installed and when temperature stability and sufficient equilibration are maintained. In the manuscript, we have clarified the main sources of uncertainty associated with the measurements, including instrumental and calibration uncertainty, temperature gradients, incomplete equilibration, condensation, sensor–leaf contact, and spatial heterogeneity in soil water potential. However, we prefer not to assign expected level to the overall uncertainty of the protocol. The effective uncertainty depends strongly on experimental conditions, sensor installation, temperature stability, equilibration, sample characteristics, and data-quality criteria. In addition, deviations from ideal psychrometer conditions, such as temperature gradients or non-equilibrium, may introduce systematic bias rather than simply increasing random measurement error. Therefore, a single uncertainty value would not adequately represent the range of conditions under which this protocol may be applied.

 There should be more detail on the 'otherwise aberrant' part in the data cleaning, and rationale should be given for removing inverted day night cycle patterns (why does this occur?).

 Answer: We thank the reviewer for this comment. We agree that the term “otherwise aberrant” was too vague and have clarified the criteria used during data cleaning in the revised manuscript. In our dataset, measurements were considered aberrant when they deviated markedly from the typical day- or night-time distribution, or when abnormal values were accompanied by diagnostic indicators from the PSY1 output, particularly unusual Wet Bulb Depression values or abnormal temperature-gradient signals (dT). These diagnostic variables were therefore used together with the water potential time series to distinguish physiologically plausible variation from likely measurement artefacts. We have also clarified the rationale for removing inverted day/night cycle patterns. Under our experimental conditions, these inverted patterns were associated with the charging/power issue described in the manuscript, which disturbed the normal measurement sequence and produced non-physiological temporal patterns in the PSY1 output. Because these patterns were linked to a technical artefact rather than to expected plant water-potential dynamics, the affected data were excluded from further analysis. This explanation has now been added to the revised manuscript (lines 564-567 and 569-570).

 The manuscript text did define the abbreviations for the legend items shown in Figure 7 for SWP but not for Tr or LWP. Figure 7 caption could have more details.

 Answer: We have revised the figure 7 legend, please check.

 It is important to caution about the use of "Measure" in the caption, because the potentials are not direct measurements, they are calculated from e.g. vapor pressure measurements using the presented theory. All three quantities should have some error bar or uncertainty estimate due to instrument error & theory error propagation.

 Answer: We thank the reviewer for this important comment. We have removed “measure” in Figure 7. We agree with the reviewer that all three quantities should have some error bar or uncertainty estimate due to instrument error & theory error propagation. As we explained to reviewer 1 and 2, Figure 7 is intended primarily as an illustrative example of PSY1 and Teros 21 use rather than as a dataset for formal statistical comparison. Because the number of biological replicates in this example was limited, we chose not to display standard deviations in Figure 7, as they would not provide a robust estimate of variability. Although a full error analysis is beyond the scope of this protocol paper, we agree that these aspects should be explicitly discussed so that users can better interpret PSY1 measurements. To clarify the importance of replication, we included the following sentence in the revised manuscript: “Because this protocol paper is intended to demonstrate PSY1 operation and interpretation, the example dataset is illustrative and not designed for rigorous statistical treatment; users should include sufficient biological replication, typically at least 3–5 replicates where possible depending on the experimental objective and biological variability, together with calibration checks and uncertainty analysis.” (line 529-534) In addition, we are currently preparing a research manuscript in which Teros 21 and PSY1 are used extensively for water potential measurements with a replication scheme suitable for formal statistical analysis.

 Figure 8 should have a legend for the blue and red line, and the caption could have more information on what issue is presented in the Figure. Answer: We added the legend and detailed the caption in the revised Figure 8, please check!

# Reviewer report: Reviewer response for version 2

**DOI:** 10.21956/openreseurope.25594.r75163

**Published:** 2026-06-22

**Authors:** Piyush Jain

**Affiliations:** 1 Cornell University, Ithaca, New York, USA

I appreciate that the authors have given due consideration to the comments and have revised the manuscript.

Is the study design appropriate for the research question?

Yes

Is the rationale for, and objectives of, the study clearly described?

Partly

Are sufficient details of the methods provided to allow replication by others?

Partly

Are the datasets clearly presented in a useable and accessible format?

Partly

Reviewer Expertise:

Plant Physiology

I confirm that I have read this submission and believe that I have an appropriate level of expertise to confirm that it is of an acceptable scientific standard.

# Reviewer report: Reviewer response for version 2

**DOI:** 10.21956/openreseurope.25594.r75164

**Published:** 2026-06-17

**Authors:** yijian zeng

**Affiliations:** 1 University of Twente, Enschede, The Netherlands

I thank the authors' efforts in revising this protocol.

Is the study design appropriate for the research question?

Yes

Is the rationale for, and objectives of, the study clearly described?

Yes

Are sufficient details of the methods provided to allow replication by others?

Yes

Are the datasets clearly presented in a useable and accessible format?

Yes

Reviewer Expertise:

soil-plant hydraulics; and its coordination with photosynthesis; soil-plant digital twin;

I confirm that I have read this submission and believe that I have an appropriate level of expertise to confirm that it is of an acceptable scientific standard.

# Reviewer report: Reviewer response for version 1

**DOI:** 10.21956/openreseurope.23128.r69654

**Published:** 2026-03-16

**Authors:** yijian zeng

**Affiliations:** 1 University of Twente, Enschede, The Netherlands

The manuscript presents a useful and timely protocol for continuous in situ monitoring of plant and soil water status using PSY1 leaf psychrometers and TEROS 21 sensors, together with a data-processing workflow and open materials on Zenodo. The practical value is clear, especially for groups that are new to continuous water-potential measurements. The step-by-step instructions, photographs, and linked code/video resources are strengths. The paper is also commendable for making the workflow accessible beyond a narrow instrumentation community.

 My main concern is that the manuscript needs a stronger treatment of measurement meaning, assumptions, and uncertainty. At present, the paper gives readers a workable installation protocol, but not yet enough guidance to ensure that the resulting measurements are interpreted correctly and reproducibly across systems.

 1. the experimental section states that TEROS 21 measures soil matric potential, while PSY1 is presented as measuring leaf water potential, including pressure and osmotic components, and the pressure chamber is discussed separately as potentially measuring either pressure potential alone or total potential.

 For physical consistency, the manuscript should explicitly state:

- what the TEROS 21 reports;
- what the PSY1 reports under equilibrium assumptions;
- under what assumptions these quantities can be compared directly;
- when osmotic effects, temperature gradients, or non-equilibrium invalidate a simple interpretation of soil-to-leaf gradients.

As written, the paper risks encouraging readers to combine Ψsoil and Ψleaf as if they were directly interchangeable without stating the required assumptions.

 2. The treatment of calibration, uncertainty, and replicate requirements is also too limited at present; directing readers to the instrument manual is not sufficient for a protocol paper.

 3. I agree with the previous reviewer that the manuscript should add a short concluding section linking the measurements back to hydraulic conductivity/conductance theory

Is the study design appropriate for the research question?

Yes

Is the rationale for, and objectives of, the study clearly described?

Yes

Are sufficient details of the methods provided to allow replication by others?

Yes

Are the datasets clearly presented in a useable and accessible format?

Yes

Reviewer Expertise:

soil-plant hydraulics; and its coordination with photosynthesis; soil-plant digital twin;

I confirm that I have read this submission and believe that I have an appropriate level of expertise to confirm that it is of an acceptable scientific standard, however I have significant reservations, as outlined above.

# Response

**Published:** 2026-05-20

**Authors:** Lei Ding

**Affiliations:** Earth and life institute, Universite catholique de Louvain, Louvain-la-Neuve, Walloon Region, Belgium

Reviewer 2 The manuscript presents a useful and timely protocol for continuous in situ monitoring of plant and soil water status using PSY1 leaf psychrometers and TEROS 21 sensors, together with a data-processing workflow and open materials on Zenodo. The practical value is clear, especially for groups that are new to continuous water-potential measurements. The step-by-step instructions, photographs, and linked code/video resources are strengths. The paper is also commendable for making the workflow accessible beyond a narrow instrumentation community.

 My main concern is that the manuscript needs a stronger treatment of measurement meaning, assumptions, and uncertainty. At present, the paper gives readers a workable installation protocol, but not yet enough guidance to ensure that the resulting measurements are interpreted correctly and reproducibly across systems.

 1. the experimental section states that TEROS 21 measures soil matric potential, while PSY1 is presented as measuring leaf water potential, including pressure and osmotic components, and the pressure chamber is discussed separately as potentially measuring either pressure potential alone or total potential.

 For physical consistency, the manuscript should explicitly state:

- what the TEROS 21 reports;
- what the PSY1 reports under equilibrium assumptions;
- under what assumptions these quantities can be compared directly;
- when osmotic effects, temperature gradients, or non-equilibrium invalidate a simple interpretation of soil-to-leaf gradients.

As written, the paper risks encouraging readers to combine Ψsoil and Ψleaf as if they were directly interchangeable without stating the required assumptions.

 Answer: We thank the reviewer for this valuable suggestion. To compare TEROS 21 and PSY1 measurements, we included comparison of both in the revised manuscript. Please check line 94-100 and Table 1 in the revised manuscript.   2. The treatment of calibration, uncertainty, and replicate requirements is also too limited at present; directing readers to the instrument manual is not sufficient for a protocol paper. Answer: We thank the reviewer for this important comment. We agree that, for a protocol paper, calibration procedures, measurement uncertainty, and replication strategy should be described explicitly rather than referring readers only to the instrument manual. As noted in our responses to Reviewer 1, we have revised the manuscript accordingly by adding a paragraph on calibration (Teros 21: line 216-222; PSY1: line 303-318) and by expanding the discussion of uncertainty associated with PSY1 measurements. To clarify the role of replication, we also added the following sentence to the revised manuscript: “Because this protocol paper is intended to demonstrate PSY1 operation and interpretation, the example dataset is illustrative and not designed for rigorous statistical treatment; users should include sufficient biological replication, typically at least 3–5 replicates where possible depending on the experimental objective and biological variability, together with calibration checks and uncertainty analysis.” (line 507-512) In addition, we are currently preparing a separate research manuscript in which Teros 21 and PSY1 is used extensively for water potential measurements with a replication scheme suitable for formal statistical analysis. We hope these revisions make the protocol more practically useful for readers seeking guidance on the proper implementation and interpretation of Teros 21 and PSY1 measurements. We have updated our code to address these errors and uncertainties to analyze the PSY1 datasets.

 3. I agree with the previous reviewer that the manuscript should add a short concluding section linking the measurements back to hydraulic conductivity/conductance theory Answer: A concluding section was included in the revised manuscript (line 550-569).

# Reviewer report: Reviewer response for version 1

**DOI:** 10.21956/openreseurope.23128.r66892

**Published:** 2026-02-17

**Authors:** Piyush Jain

**Affiliations:** 1 Cornell University, Ithaca, New York, USA

The authors present a protocol for in situ continuous monitoring of leaf and soil water potential. This contribution will help reduce the time needed to prepare and standardize water potential measurements. It is helpful for those unfamiliar with these measurements. I suggest the following additions to help readers apply this protocol more broadly:

1. Add a concluding paragraph that connects the data presented here with hydraulic conductivity measurements. The paper currently reads as two separate sections. The final measurements are not connected back to theory presented in the introduction to support the calculations.
2. State assumptions explicitly around errors inherent to psychrometry. These include temperature gradients, non-equilibrium measurements, condensation, and other issues typically encountered during these measurements.
3. Include more direction on calibrations and error analysis. Reporting standard deviations in water potential, calculating uncertainty with current methods, and determining the number of replicates needed are all critical to mitigating experimental and measurement uncertainties.

Overall, such protocols are helpful, but, they should always be accompanied with thorough presentation of pros and cons of the approach along with the uncertainties and errors associated with the measurements.

Is the study design appropriate for the research question?

Yes

Is the rationale for, and objectives of, the study clearly described?

Partly

Are sufficient details of the methods provided to allow replication by others?

Partly

Are the datasets clearly presented in a useable and accessible format?

Partly

Reviewer Expertise:

Plant Physiology

I confirm that I have read this submission and believe that I have an appropriate level of expertise to confirm that it is of an acceptable scientific standard, however I have significant reservations, as outlined above.

# Response

**Published:** 2026-05-20

**Authors:** Lei Ding

**Affiliations:** Earth and life institute, Universite catholique de Louvain, Louvain-la-Neuve, Walloon Region, Belgium

Reviewer 1 The authors present a protocol for in situ continuous monitoring of leaf and soil water potential. This contribution will help reduce the time needed to prepare and standardize water potential measurements. It is helpful for those unfamiliar with these measurements. Answer: We thank the reviewer for this positive feedback. Please find our detailed point-by-point responses below. I suggest the following additions to help readers apply this protocol more broadly: 1. Add a concluding paragraph that connects the data presented here with hydraulic conductivity measurements. The paper currently reads as two separate sections. The final measurements are not connected back to theory presented in the introduction to support the calculations. Answer: Thanks for this nice suggestion. We agree that the final measurements should be more clearly connected to the theoretical framework introduced earlier in the manuscript. Accordingly, we have added a concluding paragraph in the revised manuscript to explicitly link the presented data with hydraulic conductivity measurements and to clarify how these observations support the calculations outlined in the Introduction. Please check line 550-569 in the revised manuscript. In addition, we are currently preparing a separate manuscript that will present a full dataset generated using this protocol together with inverse modeling, allowing a more detailed quantitative interpretation of hydraulic properties. 2. State assumptions explicitly around errors inherent to psychrometry. These include temperature gradients, non-equilibrium measurements, condensation, and other issues typically encountered during these measurements. Answer: We thank the reviewer for this important suggestion. We have revised the manuscript accordingly to make the assumptions and potential sources of error inherent to psychrometric measurements more explicit. First, we now explicitly acknowledge the limitation of the PSY1 near the wet end of the water potential range (around 0 MPa), where measurement sensitivity decreases due to the underlying psychrometric principle (line 417-435). We also clarified the importance of allowing sufficient equilibration time for the leaf within the chamber, noting that this duration may vary depending on leaf conditions (line 384-397). In addition, we expanded the discussion on condensation, a common source of measurement error. We now explain how condensation can be identified in the dataset (e.g., through abnormal temperature gradients, dT), and outline practical solutions such as cleaning and reinstalling the system, as well as adjusting parameters like cooling time and chamber heating when necessary (line 417-435). Finally, we emphasized the importance of minimizing thermal gradients during measurements and included a brief discussion on proper insulation to ensure stable and reliable data acquisition (line 357-364). We have updated our code to address these errors and uncertainties to analyze the PSY1 datasets. Please check the updated code, https://doi.org/10.5281/zenodo.20080750 and revised Figures 7 and 9. 3. Include more direction on calibrations and error analysis. Reporting standard deviations in water potential, calculating uncertainty with current methods, and determining the number of replicates needed are all critical to mitigating experimental and measurement uncertainties. Answer: We thank the reviewer for this important suggestion. We agree that calibration procedures, error analysis, and replication strategy are critical for mitigating experimental and measurement uncertainties when using Teros 21 and PSY1. In the revised manuscript, we have included information about calibrations (Teros 21: line 216-222; PSY1: line 303-318), uncertainty and error analysis, especially for PSY1. In this protocol paper, Figure 7 is intended primarily as an illustrative example of PSY1 use rather than as a dataset for formal statistical comparison. Because the number of biological replicates in this example was limited, we chose not to display standard deviations in Figure 7, as they would not provide a robust estimate of variability. Although a full error analysis is beyond the scope of this protocol paper, we agree that these aspects should be explicitly discussed so that users can better interpret PSY1 measurements. To clarify the importance of replication, we included the following sentence in the revised manuscript: “Because this protocol paper is intended to demonstrate PSY1 operation and interpretation, the example dataset is illustrative and not designed for rigorous statistical treatment; users should include sufficient biological replication, typically at least 3–5 replicates where possible depending on the experimental objective and biological variability, together with calibration checks and uncertainty analysis.” (line 507-512) In addition, we are currently preparing a research manuscript in which Teros 21 and PSY1 are used extensively for water potential measurements with a replication scheme suitable for formal statistical analysis.
